# Liver stiffness measurement by magnetic resonance elastography predicts cirrhosis and decompensation in alcohol-related liver disease

**DOI:** 10.1007/s00261-024-04479-2

**Published:** 2024-07-18

**Authors:** Jingbiao Chen, Peng Xu, Kyle Kalutkiewicz, Yiyang Sheng, Fatima Warsame, Mahmoud-Adam Tahboub-Amawi, Jiahui Li, Jin Wang, Sudhakar K. Venkatesh, Richard L. Ehman, Vijay H. Shah, Douglas A. Simonetto, Meng Yin

**Affiliations:** 1 Department of Radiology, Mayo Clinic, 200 First Street SW, Rochester, MN 55905 USA; 2 Division of Gastroenterology and Hepatology, Mayo Clinic, Rochester, MN USA; 3 Department of Radiology, the Third Affiliated Hospital of Sun Yat-Sen University, Guangzhou, Guangdong China; 4 Department of Radiology, Affiliated Hospital of Xuzhou Medical University, Xuzhou, Jiangsu China

**Keywords:** Liver stiffness, MR elastography, Alcohol-related liver disease, Cirrhosis

## Abstract

**Purpose:**

To evaluate magnetic resonance elastography (MRE)—based liver stiffness measurement as a biomarker to predict the onset of cirrhosis in early-stage alcohol-related liver disease (ALD) patients, and the transition from compensated to decompensated cirrhosis in ALD.

**Methods:**

Patients with ALD and at least one MRE examination between 2007 and 2020 were included in this study. Patient demographics, liver chemistries, MELD score (within 30 days of the first MRE), and alcohol abstinence history were collected from the electronic medical records. Liver stiffness and fat fraction were measured. Disease progression was assessed in the records by noting cirrhosis onset in early-stage ALD patients and decompensation in those initially presenting with compensated cirrhosis. Nomograms and cut-off values of liver stiffness, derived from Cox proportional hazards models were created to predict the likelihood of advancing to cirrhosis or decompensation.

**Results:**

A total of 182 patients (132 men, median age 57 years) were included in this study. Among 110 patients with early-stage ALD, 23 (20.9%) developed cirrhosis after a median follow-up of 6.2 years. Among 72 patients with compensated cirrhosis, 33 (45.8%) developed decompensation after a median follow-up of 4.2 years. MRE-based liver stiffness, whether considered independently or adjusted for age, alcohol abstinence, fat fraction, and sex, was a significant and independent predictor for both future cirrhosis (Hazard ratio [HR] = 2.0–2.2, p = 0.002–0.003) and hepatic decompensation (HR = 1.2–1.3, p = 0.0001–0.006). Simplified Cox models, thresholds, and corresponding nomograms were devised for practical use, excluding non-significant or biased variables.

**Conclusions:**

MRE-based liver stiffness assessment is a useful predictor for the development of cirrhosis or decompensation in patients with ALD.

**Graphical Abstract:**

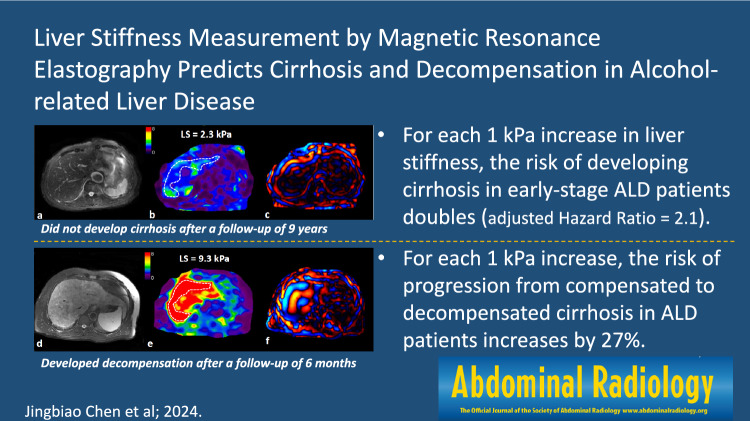

## Introduction

Alcohol-related liver disease (ALD), one of the most prevalent types of liver disease worldwide, remains a major cause of liver-related mortality, accounting for more than half of all deaths associated with chronic liver diseases [1–3].

Pathologically, ALD is a spectrum characterized by steatosis, inflammation, and fibrosis [4, 5]. In patients with biopsy-proven ALD, the annualized progression to cirrhosis was 3%, 10%, and 8%, while the annual liver-related mortalities were 1%, 7%, and 6% for patients with steatosis only, steatohepatitis only, and cirrhosis, respectively [6]. The presence of advanced fibrosis or cirrhosis in patients with compensated ALD is the main predictor of long-term survival [7]. Patients with compensated cirrhosis have a median survival of approximately nine years, compared to only about four years for those with decompensated cirrhosis [8]. However, both early-stage fibrosis and compensated liver cirrhosis are treatable. Therefore, early identification of patients at risk of developing cirrhosis or decompensation is crucial for extending survival through effective treatments.

Liver biopsy has been the reference standard for establishing a definitive diagnosis of ALD, assessing fibrosis stage, and excluding alternative causes of liver injury [9]. However, its invasive nature, suboptimal inter- and intra-observer reproducibility [10], and sampling error limit its routine application in clinical practice. Noninvasive imaging biomarkers, particularly liver stiffness with elastography, are increasingly utilized for this purpose.

Hepatic steatosis is the earliest, most common consequence of excessive alcohol consumption. Individuals with ALD-related steatosis are vulnerable to developing steatohepatitis, fibrosis, and cirrhosis. Magnetic resonance imaging (MRI) assessed fat fraction (FF) is an accurate non-invasive biomarker for quantifying hepatic steatosis, while magnetic resonance elastography (MRE) is considered the most accurate non-invasive technique for detection and staging of liver fibrosis in chronic liver diseases [11]. MRE is also useful for predicting the development of cirrhosis in noncirrhotic patients, decompensation in those with cirrhosis, and death or need for a transplant in decompensated patients [12]. Recent studies have shown that liver stiffness (LS) measured by MRE is a significant predictor of future development of cirrhosis and future decompensation or death in nonalcoholic fatty liver disease [13, 14]. Since ALD is similar to nonalcoholic fatty liver histologically, MRE may play an important role in predicting liver-related events in ALD as well.

The goal of this study was to investigate the role of MRE, in a population of ALD patients, in predicting (1) the development of cirrhosis in patients without cirrhosis at baseline, and (2) the progression from compensated to decompensated cirrhosis.

## Material and methods

### Study cohorts of ALD patients

This retrospective study was approved by the Institutional Review Board and written informed consent was waived. All patients with a history of ALD and at least one liver MRE examination between March 2007 and January 2020 were initially included in this study. Patients with alcohol-related liver disease and alcohol use disorder were identified using related ICD-9 and ICD-10 codes. Alcohol abstinence was defined as no alcohol use 90 days or greater before the MRE examination and continued until the last follow-up date or censored event date. Patients were excluded if they (1) had liver tumors or undergone liver transplantation after MRE examination; (2) follow-up duration of less than 30 days. Our research team confirmed each individual’s alcohol use history, measured quantitative imaging biomarkers (i.e., fat fraction and liver stiffness), extracted laboratory data of liver chemistries, and calculated the model for end-stage liver disease (MELD) score within 30 days of the MRE exam by manual chart review of electronic medical records (EMR).

We targeted two cohorts of patients for prognostic analyses based on their disease status:

1. ALD without cirrhosis: patients with either clinically diagnosis or biopsy-proven non-cirrhotic liver at the time of baseline MRE. A censored event for this cohort is cirrhosis development with evidence of at least one of the following: (a) stage 4 fibrosis on liver biopsy; (b) LSM ≥ 5 kPa on liver MRE; (c) cirrhotic morphology and/or signs of portal hypertension (splenomegaly or portosystemic shunting) at imaging or endoscopy (e.g., esophageal varices, portal hypertensive gastropathy), but no history of bleeding [12].
2. ALD with compensated cirrhosis: patients with a clinical diagnosis of compensated cirrhosis at the time of baseline MRE. A censored event for this cohort is at least one of the decompensation events, including esophageal bleeding, ascites, and hepatic encephalopathy. Esophageal variceal bleeding was confirmed by upper endoscopy and ascites was confirmed by imaging. Encephalopathy was recorded from clinical notes and laboratory tests [12].

### Imaging biomarkers of liver stiffness and fat fraction

All examinations were conducted on 1.5-T MR imagers (GE Healthcare). MRE examinations (two-dimensional gradient-echo or spin-echo based when appropriate) [15, 16] were performed to measure liver stiffness (LS, amplitude of complex shear modulus |G*|) after at least 4 h of fasting. Meanwhile, an estimate of hepatic fat fraction (FF) was calculated using the Dixon method from in-phase (IP) and out-of-phase (OP) gradient-recalled echo images of the liver. Readers who analyzed the LS or FF measurements were blinded to the clinical histories and laboratory results of the patients.

MRE acquisition, processing, and analysis was performed as recommended in the QIBA profile for liver MRE [17]. One reader ([M.Y.], > 20 years of experience in liver MRE) drew regions of interest (ROIs) inside the hepatic parenchyma, about 5–10 pixels away from the liver boundary, excluding regions with major blood vessels and wave confidence below 95%. The mean and standard deviation of liver stiffness in the ROIs were reported [18]. Another reader ([J.C.], > 10 years of experience in liver MRI) drew three ROIs at the hepatic hilar section on the 2-point Dixon images, including only liver parenchyma and excluding blood vessels. FF was calculated as (IP-OP)/(2 × IP), where IP is the signal intensity of the in-phase images and OP is the signal intensity of the out-of-phase images. For each subject, the mean value of the three FFs from the non-overlapped ROIs was recorded as the final FF [18, 19].

### Follow-up and clinical outcomes

Patients were followed up from the date of their first MRE examination until they developed cirrhosis, decompensation, or death. The primary outcomes were: (1) development of cirrhosis in those with early-stage ALD at baseline MRE and (2) development of decompensation in those with compensated cirrhosis at baseline MRE. All outcomes were assessed by individual chart review with team consensus, using the same definitions and criteria for compensated cirrhosis and decompensated cirrhosis as described above. Patients with less than 30 days of follow-up were excluded from our study as described above to avoid misclassification bias.

### Statistical analyses

Continuous data were expressed as median and interquartile range. Categorical data were expressed as numbers and percentages. Independent and significant parameters associated with the development of cirrhosis or decompensation were identified by the Cox proportional hazards model. A p-value of less than 0.05 was considered to be statistically significant. All statistics were performed in RStudio with the following R packages: survival (version 3.5-7) and rms (version 6.7-1).

In this study, we analyzed two distinct cohorts of ALD patients based on their liver status at the time of baseline MRE. The first cohort comprised patients with non-cirrhotic livers. For these patients, we censored the date when cirrhosis was diagnosed in subsequent follow-ups, documenting the earliest instance of such diagnosis. For cases where cirrhosis did not manifest or where the patient was lost to follow-up, the latest date available in the EMR was recorded. The second cohort consisted of patients who already had cirrhotic livers at the time of the baseline MRE. For this group, the date of the first liver decompensation event was censored. If such an event occurred, its date was recorded. In the absence of such an event, or in instances of lost follow-up, the most recent follow-up date was taken into account. It is noteworthy that a subset of ALD patients underwent multiple MREs that tracked their liver disease progression from non-cirrhosis through compensated cirrhosis and, ultimately, to decompensated cirrhosis. These patients were uniquely positioned in both cohorts, as they had two separate baseline MREs, which were utilized for prognostic analyses to predict distinct clinical events.

In our statistical methodology, we employed multivariate Cox proportional hazard models, often referred to as adjusted Cox models, to comprehensively evaluate the impact of imaging parameters (specifically, LS and FF) along with several well-established determinants known to influence the progression of ALD. These established determinants include age, considering the increased vulnerability of older individuals; sex, accounting for the increased susceptibility of females to alcohol-related liver injury; and abstinence from alcohol, a critical factor in ALD management.

In our preliminary analyses, we rigorously examined the influence of these factors on ALD progression. Only those parameters that demonstrated a statistically significant effect on the outcome were selected for inclusion in the subsequent adjusted Cox model. To maintain model parsimony and ensure robustness, we adhered to the widely accepted ‘1 out of 10’ rule of thumb in prognostic analysis. This rule recommends that, in the context of limited events, the number of predictors in the final Cox model should not exceed one-tenth of the number of observed events. Given the relatively low number of events (approximately 20–30) in our cohort, we judiciously restricted the final adjusted Cox model to include no more than three predictors. This approach strikes a balance between model complexity and statistical reliability, allowing us to capture the most influential factors while avoiding overfitting to our data. The resulting two or three-parameter Cox model represents a robust and parsimonious tool for assessing the progression of ALD within our studied population. Following the training of the Cox proportional hazards model, which demonstrated moderate accuracy and above (e.g., c-statistic > 0.65), nomograms with hazard ratios were developed using only predictors that showed a significant prognostic role.

## Results

### Study participants

**Fig. 1:**
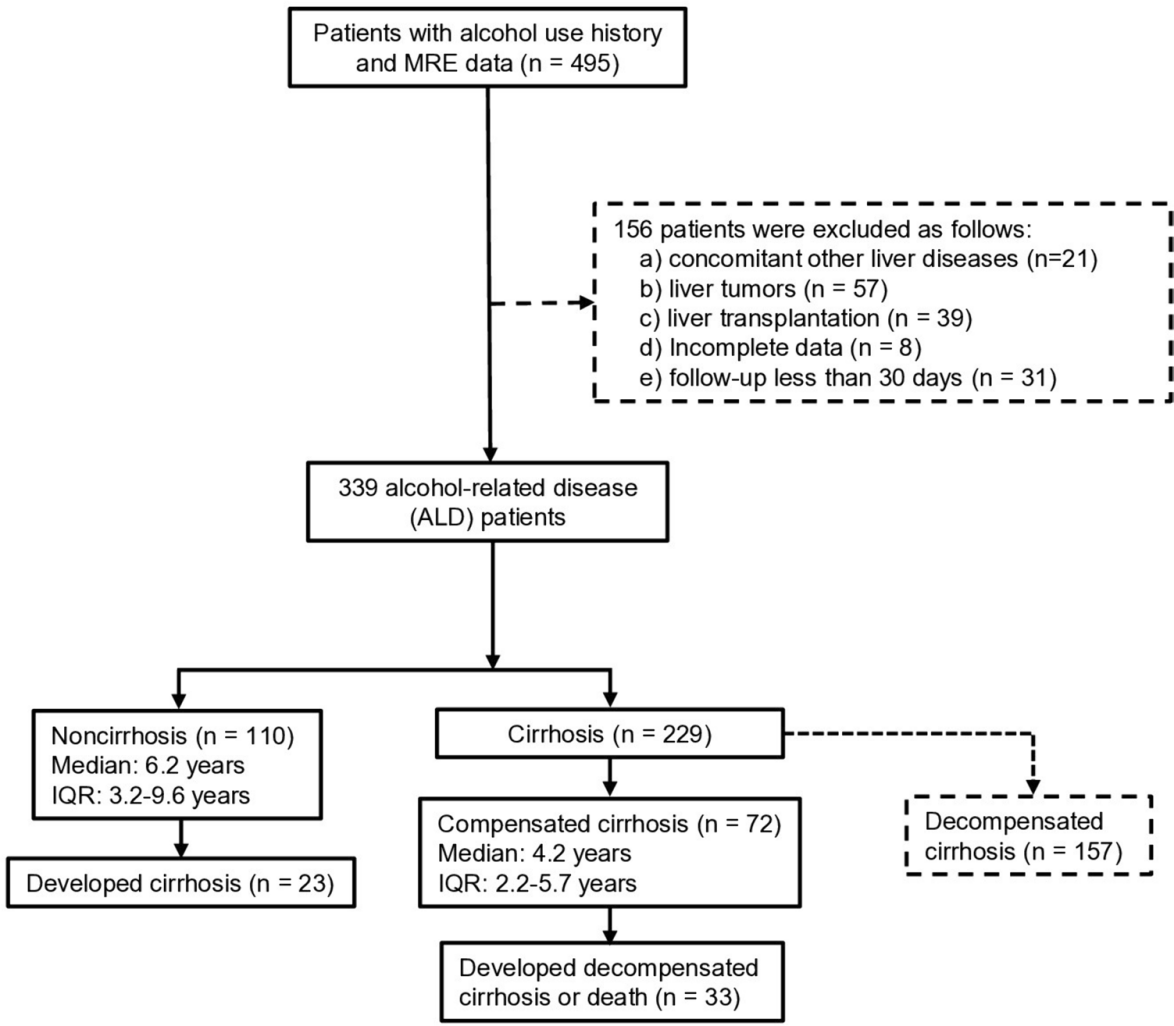
Flowchart of patient selection for the study cohort

Between March 2007 and January 2020, 495 patients with ALD and at least one liver MRE examination were initially identified. The following patients were excluded: 21 patients with concomitant other chronic liver diseases, 57 patients with liver tumors, 39 patients who had liver transplantation, 8 patients with incomplete imaging data, 31 patients with follow-up less than 30 days, and 157 patients who had decompensation at baseline (Fig. 1).

Finally, 182 patients were included in the study. Patient demographics and baseline clinical and biologic characteristics are shown in Table 1. Of the 182 patients, 132 subjects (72.5%) were males and 50 (27.5%) were females, with a median age of 57 years (interquartile range [IQR]: 51–64 years). For patient baseline CLD status, 110 patients were diagnosed as ALD without cirrhosis (noncirrhotic group) and 72 were ALD with compensated cirrhosis (compensation group) at baseline.

**Table 1:** Patient demographics and baseline clinical and biological characteristics

	Total (n = 182)	Noncirrhosis (n = 110)	Compensated cirrhosis (n = 72)
Gender*			
Male	132 (72.5%)	78 (70.9%)	54 (75.0%)
Age (years)	57 (51, 64)	56 (50, 64)	58 (52, 65)
Abstinence*	41 (22.5%)	23 (21.1%)	18 (25.0%)
Liver function tests			
AST (U/L)	52 (31, 96)	44 (28, 84)	58 (36, 98)
ALT (U/L)	54 (32, 88)	53 (30, 89)	60 (33, 79)
Total bilirubin (mg/dL)	0.7 (0.5, 1.1)	0.7 (0.4, 1.1)	0.8 (0.6, 1.2)
Albumin (g/L)	4.1 (3.7, 4.4)	4.3 (3.8, 4.5)	4.0 (3.7, 4.4)
International normalized ratio (INR)	1.0 (1.0, 1.1)	1.0 (0.9, 1.1)	1.1 (1.0, 1.2)
Platelet count (× 10^3^)	182 (139, 215)	197 (164, 243)	151 (120, 193)
MELD score	8.0 (6.4, 9.6)	7.9 (6.4, 9.3)	8.0 (7.1, 10.4)
Liver stiffness (kPa)	3.7 (2.7, 5.2)	2.9 (2.3, 3.5)	5.7 (4.8, 7.1)
Fat fraction (%)	4.7 (0.1, 16.3)	6.3 (0.3, 19.2)	2.3 (0, 11.5)
Median follow-up time (years)	4.7 (2.4, 6.9)	6.2 (3.2–9.6)	4.2 (2.2, 5.7)
Number of events*	N/A	23 (20.9%)	33 (45.8%)

Except for otherwise indicated, data are median with interquartile in the parenthesis

*Data are patient counts with the percentages in parentheses

**Fig. 2:**
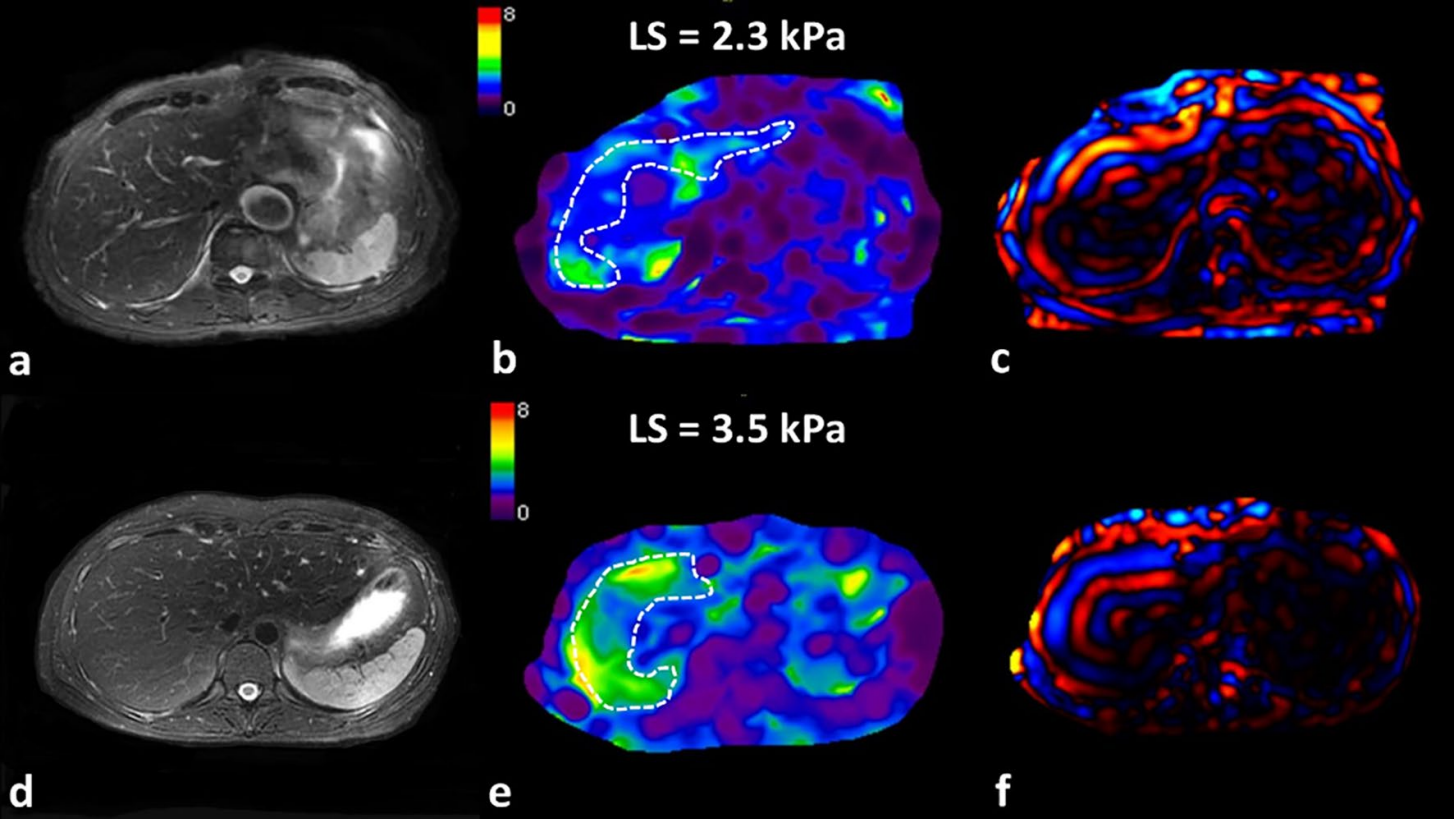
Variable progression of early-stage alcohol-related liver disease occurs with different LS levels at baseline. **a**–**c** A 69-year-old male with early-stage alcohol-related liver disease whose liver stiffness measured by MRE was 2.3 kPa at baseline. He did not develop cirrhosis after a follow-up of 9 years. **d**–**f** A 36-year-old female with early-stage alcohol-related liver disease. Her baseline liver stiffness on MRE was 3.5 kPa. She developed cirrhosis after a follow-up of 3 years. **a**, **d** T2WI; **b**, **e**: liver stiffness; **c**, **f** wave image). *LS* liver stiffness, white dashed line area, region of interest for liver stiffness measurement

In the noncirrhotic group, there were 78 (70.9%) males and 32 (29.1%) females. The median of age, liver stiffness, and FF were 56 years (IQR: 50–64 years), 2.9 kPa (IQR: 2.3–3.5 kPa), and 6.3% (IQR: 0.3–19.2%), respectively. Twenty-three (21.1%) had greater than 90 days of alcohol abstinence till the last follow-up. After a median follow-up time of 6.2 years (IQR: 3.2–9.6 years), 23 (20.9%) patients developed cirrhosis. The incidence rates of cirrhosis in the abstinence group and non-abstinence group were 13.0% (3/23) and 22.9% (20/87) respectively. Representative MRE images for two patients without cirrhosis at baseline MRE but different cirrhosis outcomes are shown in Fig. 2.

**Fig. 3:**
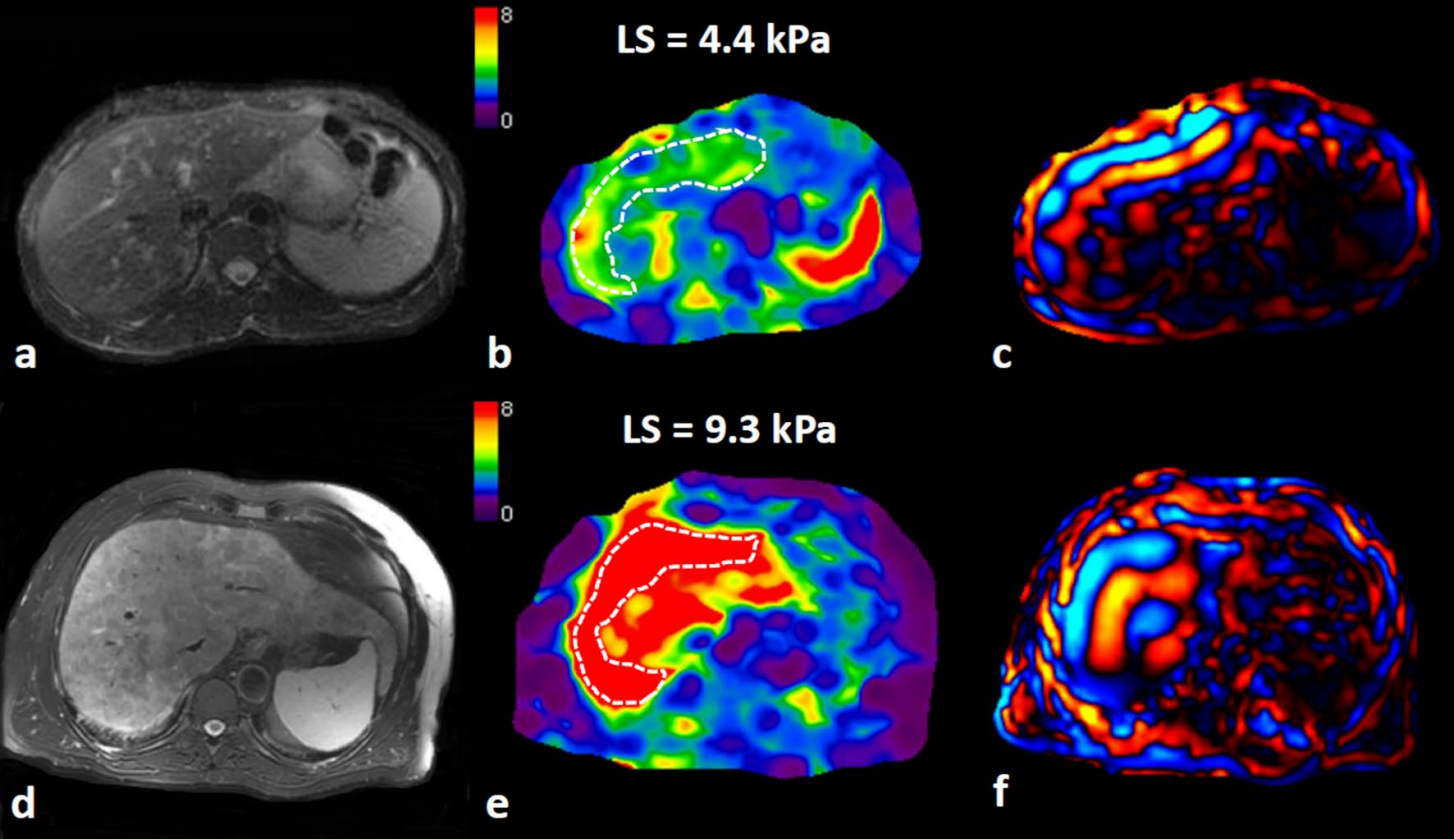
Variable progression of compensated alcoholic cirrhosis occurs with different LS levels at baseline. **a**–**c** A 48-year-old male with compensated alcoholic cirrhosis whose liver stiffness measured by MRE was 4.4 kPa at baseline. He did not develop decompensation after a follow-up of 11 years. **d**–**f** A 57-year-old male with compensated alcoholic cirrhosis. His baseline liver stiffness on MRE was 9.3 kPa. He developed decompensation after a follow-up of 6 months. **a**, **d** T2WI; **b**, **e** liver stiffness; **c**, **f** wave image). *LS* liver stiffness, white dashed line area, region of interest for liver stiffness measurement

In the compensated cirrhosis group, there were 54 (75.0%) males and 18 (25.0%) females. The median age, liver stiffness, and FF were 58 years (IQR: 52–65 years), 5.7 kPa (IQR: 4.8–7.1 kPa), and 2.3% (IQR: 0–11.5%), respectively. Eighteen of them (25.0%) had greater than 90 days of alcohol abstinence before the last follow-up. After a median follow-up time of 4.2 years (IQR: 2.2–5.7 years), 33 (45.8%) patients developed decompensation. The incidence rates of decompensated cirrhosis in the abstinence group and non-abstinence group were 33.3% (6/18) and 50.0% (27/54) respectively. Typical MRE images for patients with compensated cirrhosis at baseline MRE but different outcomes of decompensation development are shown in Fig. 3.

### Baseline liver stiffness as a predictor of cirrhosis in noncirrhotic livers

As demonstrated in Table 2, the univariate Cox model revealed that baseline liver stiffness by MRE (Hazard ratio [HR]: 2.01 per each increase of 1 kPa, p = 0.003) can be used to predict the future development of cirrhosis in noncirrhotic patients with a c-statistic of 0.67. All the other factors did not show significant prognostic value in the univariate analyses.

**Table 2:** Cox models for predicting cirrhosis in 110 ALD patients

Model	Parameter	Hazard ratio	95% CI	P-value	C-statistic
Uni-Cox	Liver stiffness (per kPa)	2.01	1.26–3.21	0.003*	0.67
Uni-Cox	FF (per 10%)	1.28	0.90–1.81	0.17	0.58
Uni-Cox	Abstinence	0.54	0.16–1.81	0.32	0.55
Uni-Cox	Age (per decade)	0.71	0.45–1.12	0.14	0.61
Uni-Cox	Sex (being male)	3.45	1.02–11.6	0.05	0.62
Multi-Cox	Liver stiffness (per kPa)	2.18	1.29–3.67	0.003*	0.77
	FF (per 10%)	1.54	1.05–2.27	0.02*	
	Abstinence	0.78	0.21–2.86	0.71	
	Age (per decade)	0.55	0.34–0.87	0.01*	
	Sex (being male)	5.34	1.39–20.5	0.01*	
Multi-Cox	Liver stiffness (per kPa)	2.10	1.31–3.36	0.002*	0.72
	FF (per 10%)	1.28	0.93–1.78	0.14	
	Age (per decade)	0.66	0.42–1.02	0.06	

*Uni-Cox* univariate Cox model, *Multi-Cox* multivariate Cox model, *FF* fat fraction

*p-value < 0.05

**Fig. 4:**
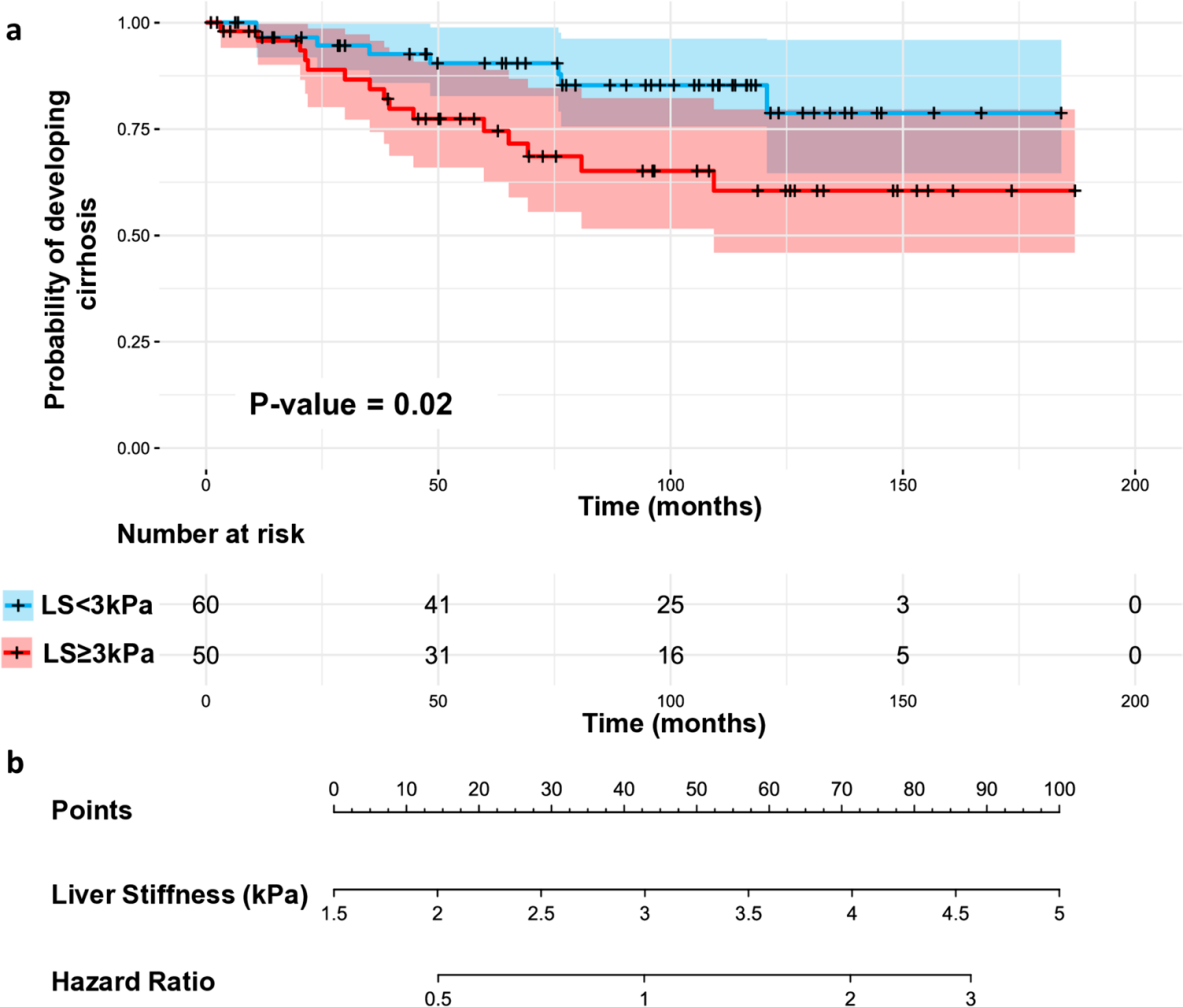
Kaplan–Meier plots for binary liver stiffness cut-off at 3 kPa (**a**) and a nomogram to predict the probability of developing cirrhosis in 110 ALD patients with non-cirrhotic livers (**b**). P-value for differences between survival curves (LS < 3 kPa vs. LS ≥ 3 kPa) was generated by the log-rank test

In multivariate analyses, after adjusting for all or two independent variables, liver stiffness was consistently shown as the most significant predictor of future cirrhosis with slightly improved overall model performance (c-statistic = 0.72–0.77). Sex was excluded from the multivariate Cox model with three predictors due to the obvious high prevalence of males (70.9%, Table 1) in this study cohort. Every 1 kPa increment in the baseline liver stiffness doubles the risk of developing cirrhosis in all Cox models. If using a categorical variable, liver stiffness ≥ 3 kPa can distinguish ALD patients with a significantly increased risk of developing cirrhosis (p = 0.02), as illustrated in the Kaplan–Meier plots of Fig. 4a for cirrhosis-free probabilities in patients with baseline liver stiffness measurements less and greater than 3 kPa, respectively. Figure 4b shows a corresponding nomogram that provides hazard ratio estimation for continuous liver stiffness measurement.

### Baseline liver stiffness as a predictor of decompensation in cirrhotic livers

As demonstrated in Table 3, both the univariate and multivariate Cox models revealed that baseline liver stiffness by MRE has a significant prognostic value (p < 0.01 for all) and can be used to predict the future development of decompensation in cirrhotic patients.

**Table 3:** Cox models for predicting decompensation in 72 cirrhotic ALD

s	Parameter	Hazard ratio	95% CI	P-value	C-statistic
Uni-Cox	Liver stiffness (per kPa)	1.20	1.05–1.39	0.006*	0.64
Uni-Cox	FF (per 10%)	0.90	0.67–1.21	0.48	0.58
Uni-Cox	Abstinence	0.59	0.24–1.43	0.24	0.52
Uni-Cox	Age (per decade)	1.28	0.89–1.82	0.18	0.56
Uni-Cox	Sex (being male)	1.98	0.76–5.14	0.16	0.56
Multi- Cox	Liver stiffness (per kPa)	1.32	1.03–1.61	0.0001*	0.69
	FF (per 10%)	0.78	0.48–1.01	0.15	
	Abstinence	0.70	0.21–1.80	0.47	
	Age (per decade)	1.43	1.07–2.87	0.08	
	Sex (being male)	2.79	0.92–13.3	0.05	
Multi- Cox	Liver stiffness (per kPa)	1.27	1.09–1.48	0.001*	0.66
	Age (per decade)	1.47	1.00–2.17	0.04*	

Likelihood ratio test for the last multi-Cox model with LS and age is 10.11 on 2 degrees of freedom and p = 0.006

*Uni-Cox* univariate Cox model, *Multi-Cox* multivariate Cox model, *FF* fat fraction

**Fig. 5:**
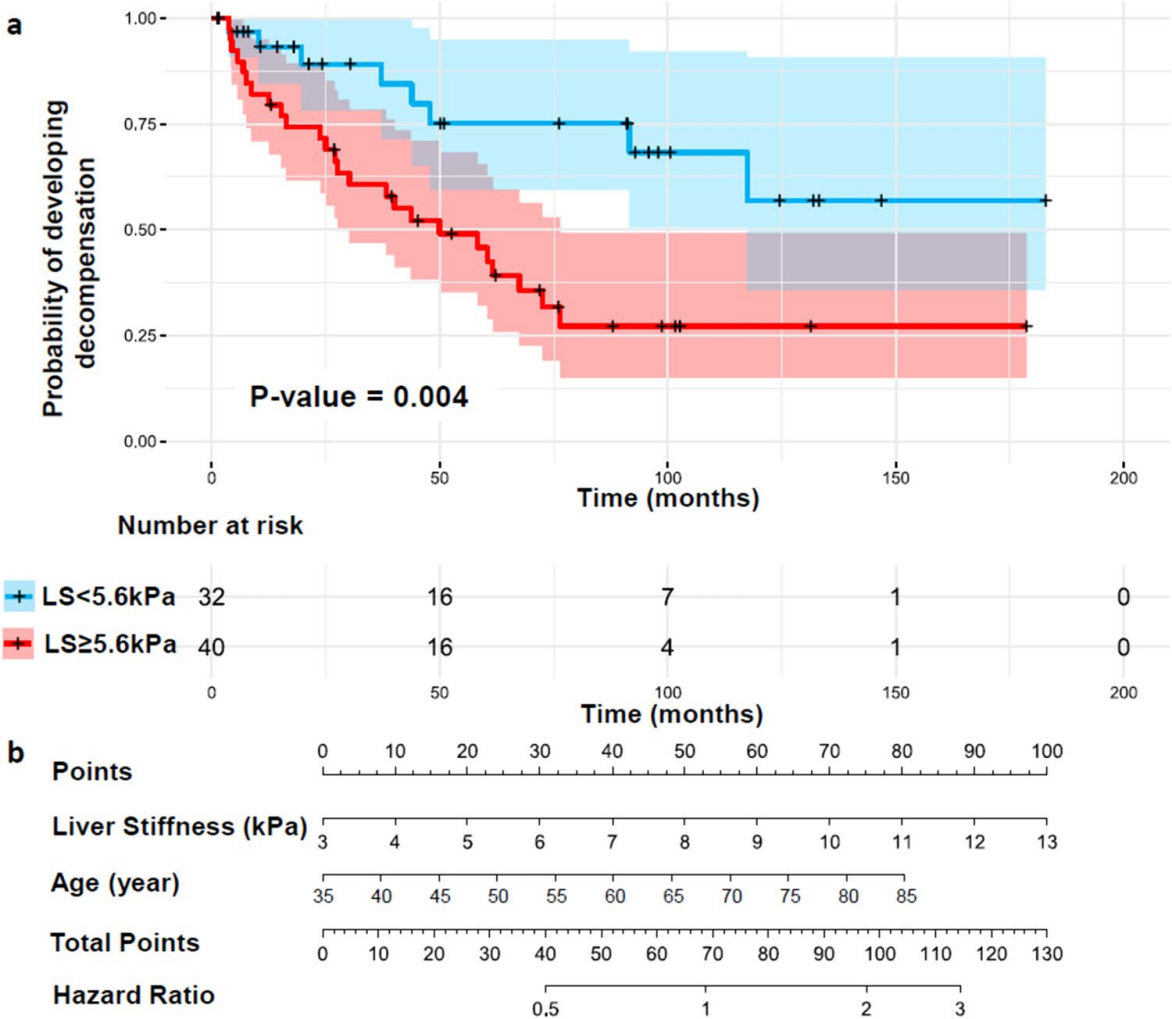
Kaplan–Meier plots for binary liver stiffness cut-off at 5.6 kPa (**a**) and a nomogram to predict the probability of developing decompensation in 72 ALD patients with compensated cirrhotic livers (**b**). P-value for differences between survival curves (LS < 5.6 kPa vs. LS ≥ 5.6 kPa) was generated by the log-rank test

When using a categorical liver stiffness cut-off at 5.6 kPa, LS ≥ 5.6 kPa could distinguish patients who have a high risk of developing decompensation (p = 0.004, Fig. 5a). After adjusting for all factors, liver stiffness by MRE was still an independent and significant predictor for future decompensation. To avoid overfitting, we kept two predictors with LS and adjusted age (per decade). Sex was excluded from analysis due to the obvious high prevalence of males (75%, Table 1) in this study cohort. The final adjusted Cox model illustrated that every 1 kPa increment in liver stiffness was associated with a 27% increased risk of developing decompensation. Age also did show significant prognostic value in this adjusted Cox model, every decade of age almost increases 50% of the risk of future compensation development. A nomogram for liver stiffness with age adjustment was provided in Fig. 5b for hazard ratio estimation.

### Prognostic values of age, fat fraction and alcohol abstinence

In our study, age emerged as a non-significant prognostic factor in predicting the onset of cirrhosis in non-cirrhotic livers. However, it showed a significant correlation with the likelihood of decompensation in patients with compensated cirrhosis. FF initially did not present as a significant factor in univariate analyses for cirrhosis or decompensation stratification. After refining the number of predictors to mitigate potential overfitting in the Cox models, FF still failed to show significant prognostic value for cirrhosis or decompensation.

Regarding alcohol abstinence, among the 23 patients in the non-cirrhotic group who reported at least 90 days of abstinence until the last follow-up, 13.0% developed cirrhosis. In the compensated cirrhosis group, 27.7% of the 18 patients reporting abstinence progressed to decompensation. Intriguingly, in our cohort, abstinence did not correlate with a reduced risk of future cirrhosis or decompensation development, as indicated in Tables 2 and 3, irrespective of adjustment in the Cox models.

## Discussion

Identifying those at high risk for the development of cirrhosis or decompensation in patients with ALD is of great clinical importance. In this retrospective, longitudinal study, liver stiffness measured by MRE alone was found to effectively predict the future development of cirrhosis in ALD patients without cirrhosis. Every 1 kPa increase in liver stiffness doubles the risk of developing cirrhosis, with a threshold of 3 kPa distinguishing those at higher risk of rapid progression. Furthermore, in patients with compensated cirrhosis, after adjusting age (per decade), each 1 kPa elevation in liver stiffness was associated with a 27% increased risk of decompensation, with 5.6 kPa serving as a critical threshold for heightened risk of rapid progression.

Our study results agree with previous studies that investigated the association between liver stiffness and future events in patients with varying chronic liver diseases. A recent study [20] explored the role of MRE in compensated and decompensated liver disease and found that liver stiffness greater than 5.8 kPa was associated with future decompensation. Other studies demonstrated that liver stiffness by MRE was a significant predictor of future development of cirrhosis, decompensation, or death in patients with metabolic dysfunction associated steatotic liver disease (previously called nonalcoholic fatty liver disease) [12–14]. In Gidener’s study [13], each 1 kPa increase in liver stiffness measured by MRE triples the likelihood of developing cirrhosis in non-cirrhotic NAFLD subjects, and elevates the risk of decompensation or death by 32% in those with NASH cirrhosis over five years. Our study reinforces these findings, demonstrating that continuous liver stiffness measurement is an independent and significant predictor of cirrhosis or decompensation in ALD. Although ultrasound-based elastography techniques, such as transient elastography and 2D-shear wave elastography, may be useful [1] as prognostic markers of different liver-related events in patients with early ALD, they may be technically limited in patients with obesity and ascites [21–23]. Compared with ultrasound-based elastography, MRE exhibits superior repeatability and reproducibility, offering the most reliable measurements of liver stiffness [24]. Additional advantages of MRE include its capacity to evaluate almost the entire liver, thereby minimizing sampling errors in the progressively heterogeneous cirrhotic liver. Moreover, MRE is highly effective in patients with obesity or ascites, where it reduces the likelihood of technical failures associated with body habitus [24].

Age per decade emerged as an independent predictor for decompensation but not cirrhosis. Intriguingly, younger ALD patients demonstrated a higher tendency towards cirrhosis [25], possibly linked to higher alcohol consumption or the recent surge in severe ALD forms in this demographic [26]. However, factors like sex bias (i.e., a high prevalence of male patients in this study cohort) led to their exclusion from our final model due to contradictory or inconsequential effects. Furthermore, this study diverges from prior research by revealing that alcohol abstinence does not emerge as a pivotal factor influencing the progression of ALD, contrasting with established findings in earlier studies [7, 27]. A limited sample size and potential abstinence reporting inaccuracies, whether intentional or unintentional, may underlie the rationale for this variance. Nonetheless, the outcomes of the present study still manifested that the occurrence of cirrhosis or decompensation remained elevated within the non-abstinence group as compared to the abstinence cohort.

Our study, while insightful, is not without limitations. First, the selection of ALD patients presents challenges. ALD often coexists with metabolic diseases due to overlapping pathophysiology and similar histological features [28]. Therefore, it is difficult to completely exclude patients with concurrent liver metabolic diseases during the selection process. Second, the study’s retrospective nature and small sample size. Its retrospective nature introduces selection bias, and the small cohort size limits the number of predictors in our Cox model. However, the strength of our study lies in the long-term follow-up and the robustness of our final regression models with fewer independent variables. Additionally, our FF measurements, despite not utilizing T2* correction, align with studies suggesting the limited prognostic value of steatosis extent. The absence of a validation cohort calls for further research to corroborate our findings.

## Conclusion

In summary, our study highlights the value of a single baseline MRE-based liver stiffness measurement in predicting cirrhosis and decompensation in patients with ALD, providing a tool for identifying high-risk individuals and guiding personalized clinical management.
